# BAM8-22 targets spinal MrgC receptors to modulate UPR^mt^ activity in the mechanism of bone cancer pain

**DOI:** 10.3389/fphar.2025.1575733

**Published:** 2025-03-31

**Authors:** Mingming Xie, Dan Li, Haohao Zeng, Yulin Huang, Rui Xu, Zhen Wang, Jiacheng Yu, Yu’e Sun

**Affiliations:** ^1^ Department of Anesthesiology, Nanjing Drum Tower Hospital, Nanjing Drum Tower Hospital Clinical College of Nanjing University of Chinese Medicine, Nanjing University of Chinese Medicine, Nanjing, China; ^2^ Department of Anesthesiology, Nanjing Drum Tower Hospital, The Affiliated Hospital of Nanjing University Medical School, Nanjing, China; ^3^ Department of Anesthesiology, Nanjing Drum Tower Hospital, Nanjing Drum Tower Hospital Clinical College of Nanjing Medical University, Nanjing Medical University, Nanjing, China

**Keywords:** bone cancer pain, Mas-related G protein-coupled receptor C, BAM8-22, mitochondrial dysfunction, mitochondrial unfolded protein response

## Abstract

**Background:**

Bone cancer pain (BCP) significantly impacts patients’ overall quality of life. Cellular energy metabolism homeostasis is critically dependent on mitochondrial integrity, and emerging evidence suggests that mitochondrial dysfunction in chronic BCP exacerbates pain progression by disrupting nociceptive signaling pathways. Notably, G protein-coupled receptors (GPCRs), a major class of membrane receptors, modulate mitochondrial function through diverse molecular mechanisms. In this study, we investigated the role of Mas-related G protein-coupled receptor C (MrgC) in BCP pathogenesis and its regulatory effects on mitochondrial function.

**Methods:**

Male C3H/HeN mice were utilized to establish a BCP model. Transmission electron microscopy and flow cytometry were employed to assess changes in mitochondrial ultrastructure, as well as levels of mtROS, ATP, and MMP in mice experiencing BCP. Following intrathecal injection of BAM8-22, we analyzed the effects of activated MrgC on mitochondrial unfolded protein response (UPR^mt^)-related molecules (ATF5, HSP60, LONP1, CLPP) and pain-related behaviors in BCP mice. The regulatory mechanism of MrgC on UPR^mt^ was further explored in N2a and 293T cells.

**Results:**

Mice with bone cancer pain showed improved mRNA and protein levels of UPR^mt^-related molecules, increased MMP and ATP, decreased mitochondrial ROS levels in the spinal cord after receiving an intrathecal injection of BAM8-22. Additionally, the paw withdrawal mechanical threshold in BCP mice increased, while the number of spontaneous foot lifts decreased. In complementary cellular studies, transfection-mediated overexpression of MrgC in N2a cells enhanced UPR^mt^ biomarker expression, whereas RNA interference-mediated MrgC knockdown produced the opposite effect.

**Conclusion:**

By activating spinal MrgC to mediate UPR^mt^ activity and protect mitochondrial function, BAM8-22 contributes to the molecular development of BCP. This discovery suggests a new therapeutic target for BCP and offers a possible research avenue.

## Introduction

Bone cancer pain (BCP) is a special pain condition resulting from the invasion of tumor cells into bone tissue, which alters the internal environment of the bone and damages surrounding nerves (Kane et al., 2015). Chronic pain symptoms affect around 75% of people with advanced cancer, with 21% specifically suffering from BCP (Coleman, 2006; Mantyh, 2013). BCP usually results from primary bone tumors or metastatic malignancies, such as renal cell carcinoma, lung cancer, thyroid cancer, prostate cancer, which spread to bones such the ribs, pelvis, tibia, and vertebrae (Clézardin et al., 2021). According to the latest 2022 data from the International Agency for Research on Cancer (IARC), there were nearly 20 million new cancer cases and approximately 9.7 million cancer-related deaths globally (Bray et al., 2024). With the rising incidence of cancer, the prevalence of BCP has increased significantly. BCP not only inflicts significant suffering on patients but also induces a range of adverse reactions, including anxiety and irritability (Sung et al., 2021), which greatly diminish their quality of life. However, the analgesic medications currently employed in clinical practice often fail to provide effective and satisfactory relief from BCP (Mantyh, 2013). Thus, there is a pressing need to identify new therapy strategies and pharmaceutical targets for BCP.

Nociceptive signaling requires substantial energy to sustain neuronal electrical propagation and synaptic transmission (Woolf and Salter, 2000; Baral et al., 2019). In the prolonged state of BCP, mitochondria undergo dynamic morphological remodeling via fission/fusion cycles to meet heightened metabolic demands (Pekkurnaz and Wang, 2022). However, excessive fission and fusion can result in mitochondrial dysfunction, which is characterized by impaired ATP production and elevated levels of reactive oxygen species (ROS) (Rambold and Pearce, 2018). These ROS exacerbate the pain response by further inducing the release of inflammatory factors (Ullah et al., 2021), while also damaging mitochondrial DNA and disrupting the proper folding of mitochondrial proteins (Fiorese et al., 2016). This cascade of events exacerbates mitochondrial damage, creating a vicious cycle. The mitochondrial unfolded protein response (UPR^mt^) serves as a critical adaptive mechanism to mitigate the detrimental effects on cells caused by protein homeostasis disruption (Shpilka and Haynes, 2018), thereby protecting cells from a broader array of mitochondrial stresses (Melber and Haynes, 2018). Therefore, improving mitochondrial activity offers a viable approach to treating bone cancer pain.

G protein-coupled receptor (GPCR) activation modulates mitochondrial function through multifaceted mechanisms (Valenzuela et al., 2021; Wu and Casey, 2024), including regulation of mitochondrial proteostasis via chaperones and proteases governing the UPR^mt^ (Izquierdo-Villalba et al., 2024). MAS-related G-protein coupled receptors (MRGR or Mrgprs) represent a class of GPCRs that are widely distributed in the central nervous system and peripheral tissues (Gour and Dong, 2024), playing roles in diverse physiological functions such as pain modulation and immune response (Cao and Roth, 2023). Studies have demonstrated that the activation of MAS receptors can impact mitochondrial function by regulating processes such as calcium ion concentration and oxidative phosphorylation (Zhang L. et al., 2024). Despite being a member of the MAS family, the mechanism of action of MrgC-particularly its influence on mitochondrial function—remains inadequately understood. BAM8-22 is anticipated to be clinically translatable as a MrgC-specific agonist with high efficacy and minimal side effects (Lv and Dong, 2010; Sun et al., 2016). This study systematically investigated mitochondrial dysfunction in BCP pathophysiology and the therapeutic effects of intrathecal BAM8-22 administration. Ultimately, we clarify how BAM8-22 contribute to the development of BCP by acting on spinal MrgC receptors to adjust UPR^mt^ activity and improve mitochondrial function.

## Materials and methods

### Animals

All animal studies followed the ARRIVE guidelines. The Experimental Animal Ethics Committee of Nanjing Hospital, affiliated with Nanjing Medical University, approved the experimental protocol (Ethical Approval No. 82071229). This study utilized 4–5 weeks old adult male C3H/HeNCrl mice obtained from Beijing Viton Lihua Laboratory Animal Technology Co., Ltd. (Beijing, China). The animals were housed under controlled environmental conditions, including a temperature-regulated (23°C ± 1°C) and humidity-controlled (50% ± 10%) specific pathogen-free (SPF) facility with a 12-h photoperiod cycle. Throughout the experimental period, the mice had *ad libitum* access to standard rodent chow and water.

We performed four experiments to elucidate the protective effect of BAM8-22 activation of MrgC receptors on spinal cord mitochondrial function. Firstly, we randomly divided the mice into bone cancer pain group (BCP) and sham operation group (Sham), 6-8 mice in each group, and spinal cord tissues were taken on the 21st day after modeling for mitochondrial function assessment. Secondly, we randomly divided the mice into BCP + Vehicle group and BCP + BAM8-22 group, 6-8 mice in each group, on the 21st day after modeling, intrathecal injection of BAM8-22 and physiological saline was carried out. Six hours after injection, spinal cord tissue was collected for mitochondrial function assessment and to detect changes in the expression of UPR^mt^-related proteins using PCR and Western blot methods. Third, the mice were randomly allocated into the Sham + Vehicle group, Sham + BAM8-22 group, BCP + Vehicle group, and BCP + BAM8-22 group, with 4 mice in each group, and on the 21st day after the modeling, intrathecal injections of BAM8-22 and saline were administered, and spinal cord tissues were taken for mitochondrial function analysis after 6 h (the Department of results are shown in Supplementary Figure 1). Fourth, we randomly divided the mice into the Sham + Vehicle group, Sham + BAM8-22 group, BCP + Vehicle group, and BCP + BAM8-22 group, 4 mice in each group. On the 21st day after the modeling, the intrathecal injection of BAM8-22 and saline was performed, and the intrathecal injection of MitoSOX was performed after 6 h. Seventy minutes post-intervention, spinal cord tissues were harvested for fluorescence Ran staining analysis.

All experimental animals were humanely anesthetized using institutionally approved protocols, and the success of the modeling was judged based on behavioral tests and tumor growth to ensure that the basic rights of the animals were respected and protected. Strict adherence to the 3R principles (Replacement, Reduction, Refinement) was maintained throughout, with daily welfare monitoring to mitigate procedure-related distress.

### Cell culture, siRNA, and plasmid transfection

Mouse fibrosarcoma cells NCTC2472 (American Type Culture Collection, CCL-11, RRID: CVCL_3067), mouse brain neuroma cells Neuro-2a (Shanghai Fuheng Biotechnology Co., Ltd., FH0424, RRID: CVCL_0470) and human embryonic kidney cells 293T (Shanghai Fuheng Biotechnology Co., Ltd., FH0244, RRID: ACC-635) were cultivated in high-sugar DMEM (VivaCell, Italy), enhanced with 100 U/mL of Gibco penicillin, 100 μg/mL of Gibco streptomycin, and 10% fetal bovine serum (Gibco, USA). The cells were kept at 37°C in an environment with 5% CO2 (Thermo Forma, USA). The experiments investigating cellular drug delivery were classified into two distinct groups: a control group cultured with DMEM, and a BAMB-22 group, which was treated with BAM8-22 (10 μM, MedChemExpress). Following a 48-h incubation period, cellular proteins were harvested for further experiments. Twenty-four hours prior to the transfection procedure, cells were planted in 6-well plates at 50% confluence. For transfection, Lipofectamine 3000 (L3000015, Thermo Fisher Technology Co., Ltd.) was employed according to the manufacturer’s guidelines. Each well was supplied with 1.5 mL of antibiotic-free medium, comprising 5 μL/mL of Lipofectamine and either 100 nM siRNA (Shanghai Gemma Pharmaceuticals Co., Ltd., target sequence 5′-CCC​AUA​AAU​AUA​AGC​AAA​GAT​T-3′) or 3 μg/mL of plasmid (IDs: 404242, 259249, Ubiquitin). The cultures were kept at 37°C for a duration of 48–72 h, with the medium being refreshed as necessary based on cell condition, after which proteins were collected for subsequent analyses.

### Cell STR authentication and *Mycoplasma* testing

Neuro-2a (Shanghai Fuheng Biotechnology Co., Ltd., FH0424, RRID: CVCL_0470) and 293T cells (Shanghai Fuheng Biotechnology Co., Ltd., FH0244, RRID: ACC-635) were cultured in a cell culture incubator. Once adherent growth was complete, the precipitate was collected through digestion and centrifugation for short tandem repeat (STR) identification and *mycoplasma* detection.

STR identification is a technique used to ascertain the true identity of cells. Axygen’s genome extraction kit was used to extract the DNA from the cell pellet, and the 21-STR amplification methodology was then used to amplify it. The ABI 3730XL genetic analyzer was used to find the STR loci and the gender gene Amelogenin. Finally, the obtained data were compared with the standard STR pattern to confirm cell identity. To detect potential *mycoplasma* contamination, we employed fluorescence quantitative PCR. Utilizing the fluorescence quantitative PCR platform, the fluorescent dye SYBR GREEN I was applied to quantitatively detect *mycoplasma* DNA in a single reaction.

### Establishment of mouse model of BCP and drug administration

The methodology presented by Schwei et al. (Schwei et al., 1999) laid the groundwork for the creation of the BCP model. For anesthesia, a 50 mg/kg dose of sodium pentobarbital was administered intraperitoneally to the animals. After achieving general anesthesia, an incision was made in the right knee. 20 μL of α-minimum essential medium (Thermo Fisher Scientific, USA) containing 2 × 10^5 NCTC 2472 cells was then added to the right femur’s intramedullary cavity; in contrast, the sham-operated group received 20 µL of α-minimum essential medium alone. Following a 21-day period designated for modeling, intrathecal injections were performed utilizing a 10-µL microsyringe, which was placed into the L5–L6 vertebral space of the mice. Reflexive tail wagging suggested successful penetration into the myelin sheath. The MrgC agonist BAM8-22 powder was first reconstituted in 0.9% saline and then diluted with additional 0.9% saline prior to administration. Animals in the BCP + BAM8-22 group were administered intrathecal injections of BAM8-22 (8.0 nM, 5 µL), whereas the BCP + Vehicle group received an equivalent volume of vehicle solution.

### Nociceptive behavior test

Tests were assessed in mice prior to the operation (day 0) and again on days 1, 4, 7, 10, 14, 21, and 28 post-operation for each group, conducted 6 h after the intrathecal administration of either BAM8-22 or vehicle. To ensure the integrity of the results, the group assignments were hidden from every researcher who conducted the behavioral tests.

#### Paw withdrawal mechanical threshold

As outlined in earlier studies, the mechanical withdrawal thresholds of the right hind paw were assessed using a series of von Frey filaments (0.16, 0.4, 0.6, 1.0, 1.4, 2.0 g; Stoelting, USA) combined with the up-down paradigm (Bonin et al., 2014). During a 30-min acclimatization phase, mice were kept in transparent compartments made of plexiglass with wire mesh bottoms. After this period, von Frey filaments were applied perpendicularly to the plantar surface, and the minimal filament force required to elicit a paw withdrawal or flinching response was documented.

#### Number of spontaneous flinches

Mice were housed in a transparent Plexiglas enclosure (10 cm × 10 cm × 15 cm) that had a wire mesh floor. Following a 30-min acclimation period, the frequency with which each mouse’s right hind limb spontaneously lifted its foot (NSF) was recorded over a 2-min timeframe. Each mouse underwent five measurements, and the average NSF value was noted.

### Transmission electron microscopy (TEM) assay

A spinal cord lumbar expansion of 1 mm^3^ was excised and stored in 2.5% glutaraldehyde at 4°C. After that, the spinal cord was dehydrated using an ethanol gradient, preserved from light at room temperature, and fixed in 1% osmium tetroxide. Ultrathin 70 nm sections were embedded in epoxy resin, cut, and placed on a 200-mesh copper grid. Uranyl acetate and lead citrate were then used to stain the sections. Using a Hitachi electron microscope, pictures were taken.

### Measurement of mitochondrial ROS generation

Following anesthesia induction, the lumbar spinal cord segments were dissected and enzymatically dissociated in six-well plates to generate single-cell suspensions. Subsequently, 200 μL aliquots of the cell suspension were combined with 10 μM DCFH-DA (S0033S, Beyotime, China) and maintained at 37°C in a cell culture incubator for 20 min. Following the removal of the supernatant from the incubation, the pellet was centrifuged and resuspended in PBS after being cleaned three times with 1640 vacuolar solution. The final samples were subsequently analyzed utilizing a flow cytometer along with FlowJo software.

### Measurement of MMP

The evaluation of MMP was conducted utilizing the fluorescent probe JC-1 (C2001S, Beyotime, China). A collection of between 100,000 and 600,000 cells was made and resuspended in 0.5 mL of cell culture medium. Following this, 0.5 mL of the working solution for JC-1 staining was incorporated, and the resulting mixture was gently inverted multiple times to achieve uniform mixing. The cells were maintained at 37°C in a cell culture incubator for 20 min. After incubation, the samples were centrifuged at 600g for 3–4 min at 4°C, during which the supernatant was carefully removed. Following centrifugation, the pellet was resuspended in PBS after being washed twice with JC-1 staining buffer (1X). Subsequently, the samples were analyzed using flow cytometry and processed with FlowJo software.

### Measurement of ATP content

ATP quantification was performed using a commercial ATP Assay Kit (S0026, Beyotime) following manufacturer specifications. Tissue lysates were prepared at a 1:10 (w/v) ratio in ice-cold lysis buffer. Homogenates were centrifuged at 12,000 × g for 5 min at 4°C, and supernatants were collected for analysis. Aliquots (20 μL) of supernatant were mixed with 100 μL ATP working solution in 96-well plates, incubated for 3–5 min at 25°C, the measurement of the relative light unit (RLU) value or counts per minute (CPM) was conducted utilizing either a chemiluminescence luminometer or a liquid flash meter.

### Mitochondrial superoxide image in the SCDH

We assessed mitochondrial ROS generation with the MitoSOX Red reagent (M36008, Invitrogen, Shanghai, China), a well-known marker for mitochondrial superoxide as reported in previous investigations (Sun et al., 2022). MitoSOX Red was dissolved in a solution comprising 2% DMSO in saline before being adjusted to a final concentration of 33 μM, as described in reference (Chen et al., 2024). A total of 10 µL of MitoSOX Red was intrathecally administrated to mice. Approximately 70 min after the injection, the animals underwent intracardiac perfusion with 4% paraformaldehyde dissolved in 0.1 M PBS for tissue sampling. After being collected, the L3–L5 spinal cord segments were implanted and cut into 20 µm-thick slices. Next, the NeuN antibody (1:1000, Novus Biologicals, NBP3-05554) was incubated on these tissue sections. Using a fluorescent microscope (BX53, Olympus, Japan), imaging was done to get pictures that showed mitochondrial superoxide. The percentage of immunostaining was measured utilizing ImageJ software. All experiments were conducted under conditions devoid of light.

### Western blotting

The mice were deeply anesthetized with pentobarbital (50 mg/kg, delivered intraperitoneally) and euthanized at 21 days after surgery, 6 h post-drug administration. The M-PER Mammalian Protein Extraction Reagent (ThermoFisher) was used to extract proteins from the ipsilateral portions of the L3-L5 spinal cord. Protein quantification was performed using the BCA Protein Assay Kit. Anti-MrgC (1:1000; Invitrogen, PA5-89019), anti-β-Actin (1:20,000; Bioss, bs-0061R), anti-ATF5 (1:2000; Abcam, ab184923), anti-HSP60 (1:20,000; Proteintech, 66041-1-Ig), anti-LONP1 (1:5000; Proteintech, 66043-1-Ig), and anti-CLPP (1:2000; Proteintech, 66271-1-Ig) were the main antibodies utilized. Secondary antibodies included goat anti-rabbit (1:2000; Beyotime, A0208) and goat anti-mouse (1:2000; Beyotime, A0216) antibodies. An enhanced chemiluminescence solution (Vazyme) was used to detect protein bands, viewed with a cooled charge-coupled device system (Tanon) and examined with ImageJ software.

### Real-time quantitative PCR

Total RNA was extracted from the mouse spinal cords using Trizol reagent (Invitrogen, USA). The reverse transcription was performed using a Vazyme cDNA reverse transcription kit (China). The primer design is shown in Table 1. The PCR protocol consisted of a melting curve analysis with steps at 95°C for 15 s, 60°C for 60 s, and 95°C for 15 s, followed by an initial denaturation at 95°C for 30 s and cycling conditions of 95°C for 10 s and 60°C for 30 s. Data analysis was performed using the 2^(−ΔΔCT)^ method.

**TABLE 1:** Primer sequences for real-time quantitative PCR.

Gene	Forward primer (5'–3′)	Reverse primer (5′–3′)
*ACTB* *ATF5*	CTACCTCATGAAGATCCTGACC ACCGCAAGCAAAAGAAGAGA	CACAGCTTCTCTTTGATGTCAC CAGCCTGGGACCTGTACCCTA
*HSP60*	TGG​GGT​CAC​TGT​TGC​AAA​GT	ATC​CCC​AGC​CTC​TTC​GTT​TG
*CLPP*	CCA​TCC​AGG​CAG​AGG​AAA​TCA	CTC​TCC​ATT​GCT​GAC​TCG​AT
*LONP1*	ACA​AGA​TTG​GGC​CGA​GGC​TAC	CGT​GCA​GAT​GAA​TAG​CAC​CTT​G

### Hematoxylin and eosin staining

After being fixed in 10% paraformaldehyde and decalcified in an EDTA solution for one to 2 weeks, mouse femur tissues were embedded in paraffin. They were subsequently dehydrated, cut into slices that were 5 μm thick, regularly stained with hematoxylin-eosin, and photographed using an Olympus laser scanning confocal microscope.

### Statistical analysis

Version 9.0.0 of GraphPad Prism was used for statistical analyses and visual displays. Every data point is displayed as mean ± standard deviation. The study employed repeated measures analysis of variance (ANOVA) to investigate changes in harmful behavior within groups over time, and multiple comparisons to evaluate differences in hazardous behaviors across groups at each time interval. The significance of the differences was evaluated using Bonferroni *post hoc* testing. For data exhibiting uniform variance and following a normal distribution, as determined by RT-PCR and Western blotting, we compared the two groups using an independent samples t-test. Statistical significance was set at p < 0.05 for all two-tailed analyses. Sample sizes for each experiment were determined based on our previous studies (Wang et al., 2020).

## Results

### In the BCP group, there is destruction of bone tissues, a reduction in the mechanical pain threshold, and an increase in spontaneous flinching

**FIGURE 1:**
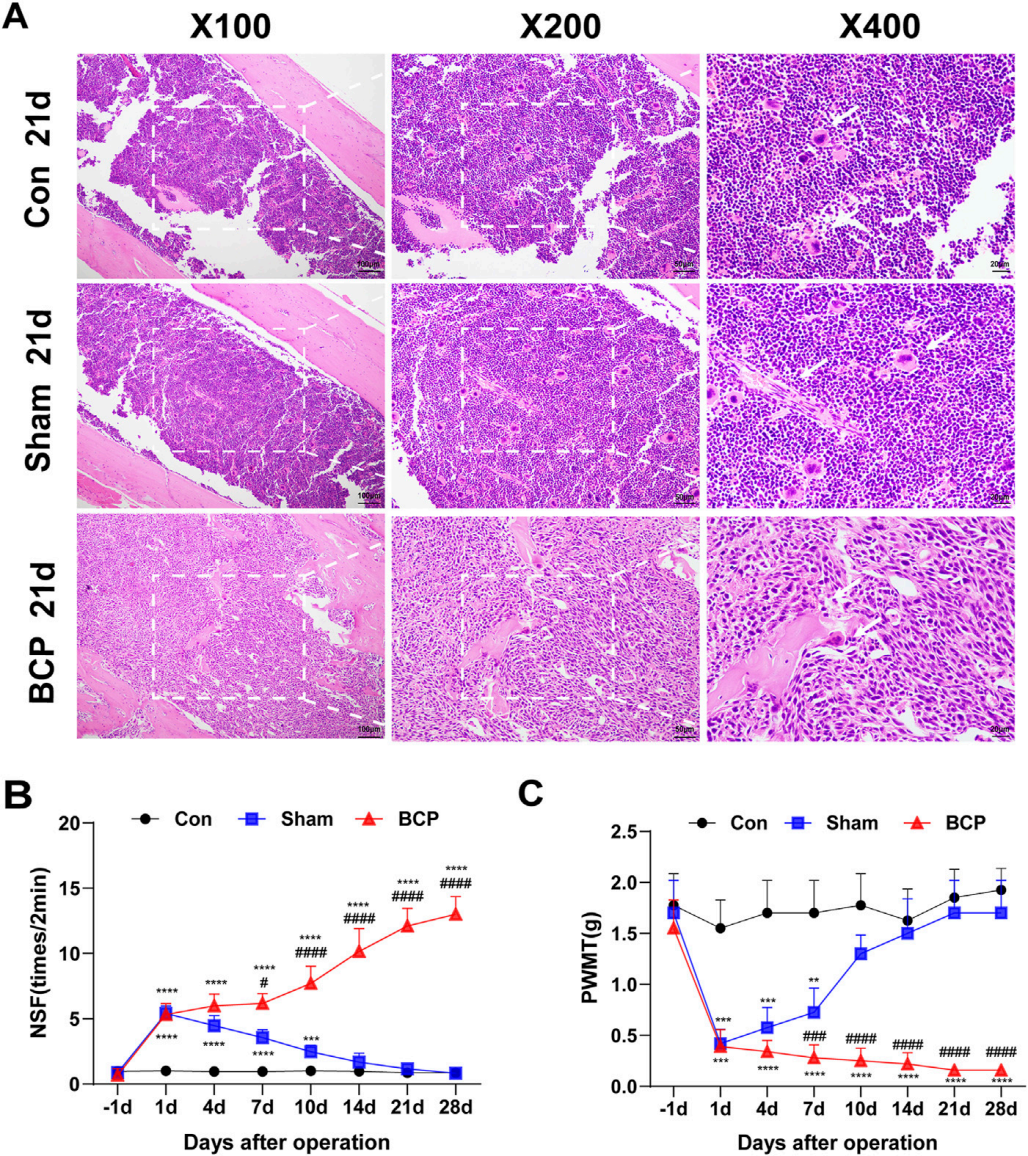
HE staining and behavioral changes in mouse femur tissue. **(A)** In the Control, BCP, and Sham groups, microscopic pictures of the femoral bone marrow cavity stained with hematoxylin and eosin on postoperative day 21 (magnifications:×100, ×200, ×400). **(B, C)** Preoperative day 1 (baseline) and 1, 4, 7, 10, 14, 21, and 28 days postoperatively were used to measure the PWMT and the NSF in the Control, BCP, and Sham groups. Statistical analyses were performed using one-way ANOVA followed by Bonferroni *post hoc* tests (*P < 0.05, **P < 0.01, ***P < 0.001 versus day 0) and two-way repeated-measures ANOVA with Bonferroni correction (#P < 0.05, ##P < 0.01, ###P < 0.001 versus sham group at corresponding time points). N = 8 per group. Data are expressed as mean ± SD.

NCTC 2472 fibrosarcoma cells were injected into the right femur of mice to produce a BCP model. To check for tumor growth and bone loss in the femur’s bone marrow cavity, HE staining was performed after 21 days. Mice in the BCP group showed tumor cell infiltration, bone trabeculae breakage, bone tissue degradation, and bone marrow cell loss in their bone marrow cavities when compared to the Sham group (Figure 1A). Pain behavioral changes in the mice were assessed by measuring the nociceptive responses using the PWMT and NSF on preoperative day 1 and postoperative days 1, 4, 7, 10, 14, 21, and 28. There were no discernible variations in NSF or PWMT between the BCP and Sham groups on the first preoperative day. On the first postoperative day, however, PWMT dropped and NSF rose in both groups in comparison to the preoperative period (Figures 1B,C). The mice in the Sham group thereafter gradually reverted to their preoperative pain behaviors (Figures 1B,C). In contrast, mice in the BCP group showed a gradual decrease in PWMT and a gradual increase in NSF over time (Figures 1B,C), suggesting that mice developed persistent bone cancer pain.

### Mitochondrial dysfunction in the spinal cord of mice in the state of BCP

**FIGURE 2:**
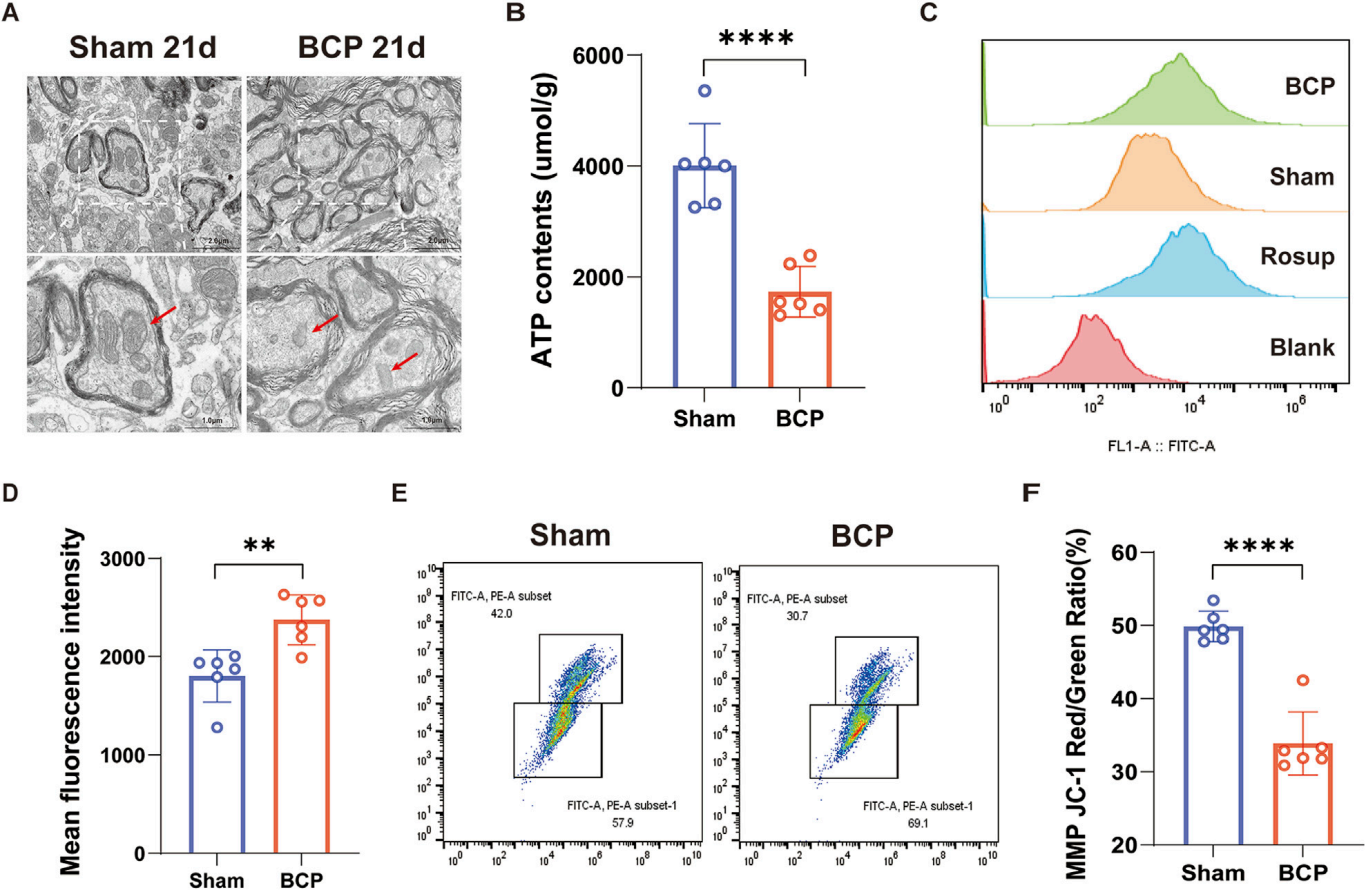
Mitochondrial morphology in the spinal cord of mice with BCP is disrupted, characterized by decreased ATP content, increased ROS production, and reduced MMP. **(A)** Transmission electron microscopy reveals ultrastructural changes in spinal cord mitochondria on day 21 postoperatively. Scale bars are set at 2.0 μm (left) and 1.0 μm (right). **(B)** Spinal cord mitochondrial ATP levels on postoperative day 21 are shown. **(C, D)** Quantitative plots and flow histograms show the average fluorescence intensity of mtROS in spinal cord tissue on day 21 after surgery. **(E, F)** Quantitative maps and flow scatter plots show the spinal cord’s mitochondrial MMP on day 21 after surgery. An independent t-test was used to determine statistical significance (*p < 0.05, **p < 0.01, ***p < 0.001 in comparison to the sham group; n = 6 per group).

Changes in the mitochondria of the spinal cord in mice were examined using transmission electron microscopy. Compared to the Sham group, the BCP group exhibited enlarged mitochondria within their spinal cords, characterized by a blurred or completely absent cristae structure (Figure 2A). The content of intracellular ATP is a vital measure of mitochondrial functionality (Boyman et al., 2020); in comparison to the Sham group, the BCP group’s mice showed noticeably lower levels of ATP in their spinal cord mitochondria (Figure 2B). ROS are pivotal mediators of cellular damage, primarily through their ability to diminish mitochondrial membrane potential, thereby impairing ATP synthesis. A self-amplifying cycle of mitochondrial permeability transition is started when pathological ROS buildup directly causes the mitochondrial permeability transition pore (mPTP) to open continuously. This process significantly worsens structural and functional impairments within the mitochondria (Bravo et al., 2024). Notably, ROS levels were significantly elevated in the spinal cord of mice experiencing bone cancer pain compared to the Sham group (Figures 2C, D). Mitochondrial Membrane Potential (MMP) reflects the integrity and functional status of mitochondria (Morse et al., 2024). Using a JC-1 fluorescent probe, we measured the amount of mitochondrial membrane potential in the mice’s spinal cords. The results showed that the BCP group’s MMP was much lower than the Sham group’s (Figures 2E, F). In conclusion, mice suffering from bone cancer showed substantial changes in the ultrastructure and activity of mitochondria in their spinal cords.

### BAM8-22 improves mitochondrial function in mouse spinal cord and alleviates bone cancer pain in mice

**FIGURE 3:**
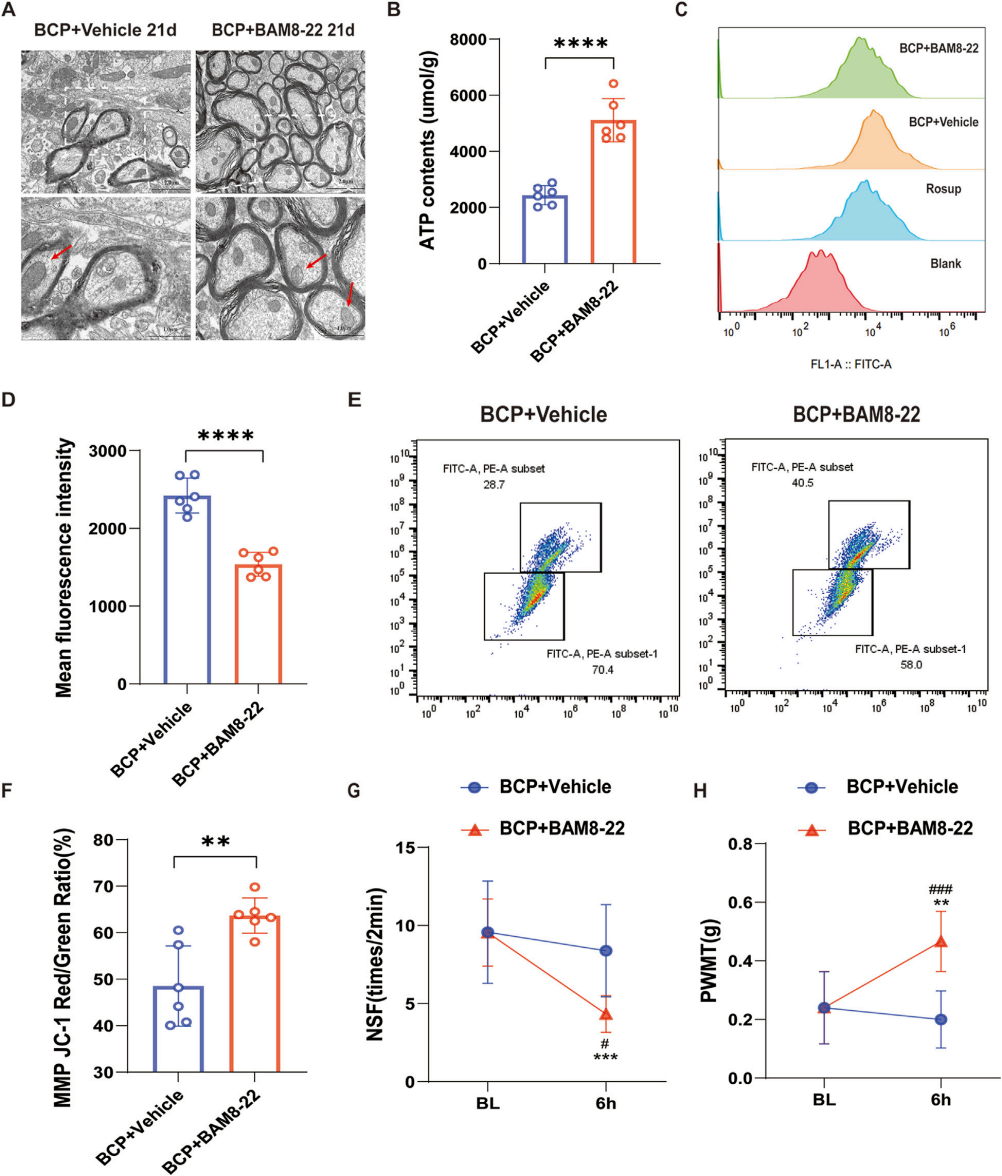
Effect of BAM8-22 on mitochondrial function. **(A)** Transmission electron microscopy observations reveal ultrastructural changes in spinal cord mitochondria, with scale bars measuring 2.0 µm (left) and 1.0 µm (right). **(B)** Mitochondrial ATP levels in the spinal cord are presented. **(C, D)** Flow histograms and quantitative plots illustrate the mean fluorescence intensity of mitochondrial reactive oxygen species (mtROS) in spinal cord tissue. **(E, F)** Flow scatter plots and quantitative maps depict mitochondrial membrane potential (MMP) in the spinal cord. **(G, H)** The PWMT in the right hind limb of mice and the NSF were evaluated 6 hours following the intrathecal administration of BAM8-22 and 21 days post-surgery. An independent t-test was utilized for statistical analysis in comparison to the solvent group (significance levels were indicated as *p < 0.05, **p < 0.01, and ***p < 0.001; n = 6 per group).

BAM8-22 specifically agonizes the MrgC receptor (He et al., 2014). We injected BAM8-22 intrathecally into mice 21 days after surgery to investigate the effect of MrgC activation on mitochondrial activity. Six hours post-intervention, spinal cord tissue specimens were collected for analysis of mitochondrial morphology and functional integrity. The mouse spinal cord’s mitochondrial ultrastructure was improved after BAM8-22 therapy as compared to the solvent control. This finding was supported by enhanced mitochondrial cristae structure, a more homogeneous morphology, and a reduction in mitochondrial volume (Figure 3A). Additionally, following intrathecal BAM8-22 treatment, we saw a substantial increase in spinal cord ATP levels (Figure 3B) and a decrease in ROS levels (Figures 3C, D), and mitochondrial membrane potential was notably increased (Figures 3E, F). Six hours after BAM8-22 administration, behavioral pain assessments showed a large decrease in NSF and a marked increase in PWMT as compared to pre-administration tests (Figures 3G, H). On the other hand, mice in the solvent group showed no appreciable alterations in their pain behavior (Figures 3G, H). In conclusion, BAM8-22 reduced pain perception in the setting of BCP and enhanced the structure and function of spinal cord mitochondria.

### Intrathecal injection of BAM8-22 attenuates spinal cord mitochondrial superoxide expression and protects spinal cord mitochondrial function

**FIGURE 4:**
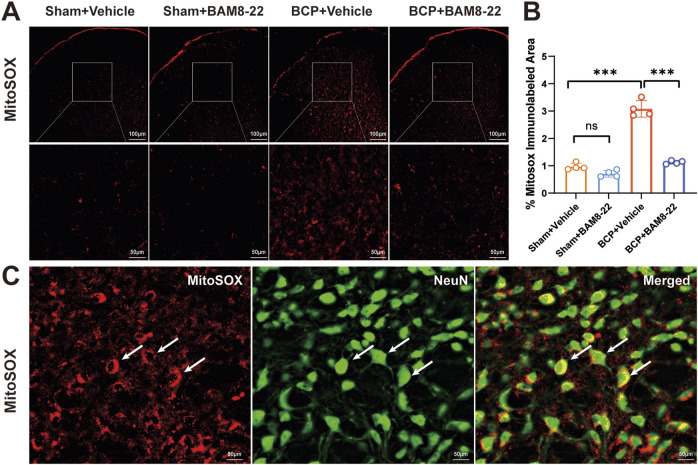
Intrathecal injection of BAM8-22 improved mitochondrial function in the dorsal horn of the spinal cord of BCP mice. **(A)** Representative images depicting MitoSOX in the spinal cord dorsal horn (SCDH) of mice across various groups. Scale bars: 100µm and 50 µm. **(B)** Mice from the BCP + Vehicle group showed significantly more MitoSOX immunoreactivity in their SCDH compared to the Sham + Vehicle group, according to quantitative analysis. When BAM8-22 was administered to BCP mice, MitoSOX immunoreactivity was decreased. **(C)** The picture shows that NeuN (shown by the arrow) co-localized with MitoSOX-positive cells. (*p < 0.05, **p < 0.01, ***p < 0.001 vs. respective groups; n = 4 per group).

Extensive research has demonstrated that mitochondrial dysfunction serves as a key mechanism contributing to both the development and maintenance of chronic pain (Shao et al., 2021). Chronic pain is closely linked to the presence of mitochondrial reactive oxygen species (MitoROS), which are crucial in activating and sustaining the signaling pathways involved in chronic pain development. Compelling evidence from neuropathic/inflammatory pain models reveals selective MitoROS accumulation within glutamatergic spinal cord dorsal horn (SCDH) neurons (Godai et al., 2019; Chen et al., 2024). Researchers used MitoSOX imaging to measure mitochondrial superoxide levels in the L3-L5 areas of the spinal cord in a rat model of BCP in order to clarify the function of spinal ROS. The MitoSOX Red reagent was used to measure the production of ROS in order to evaluate mitochondrial function. When comparing the BCP + Vehicle group to the Sham + Vehicle group, the results showed a considerable increase in MitoSOX immunoreactivity, suggesting a major impairment of mitochondrial function within the BCP mice’s spinal cords (Figures 4A, B). Interestingly, after BAM8-22 was administered by intrathecal injection, the BCP + BAM8-22 group’s MitoSOX immunoreactivity was much lower than that of the BCP + Vehicle group (Figures 4A, B). This discovery raises the possibility that BAM8-22 may safeguard spinal cord mitochondrial activity, indicating a potential therapeutic pathway for alleviating mitochondrial dysfunction associated with chronic pain. Furthermore, nearly all MitoSOX signals were found to colocalize with NeuN (Figure 4C).

### Intrathecal injection of BAM8-22 increases spinal cord UPR^mt^ levels

**FIGURE 5:**
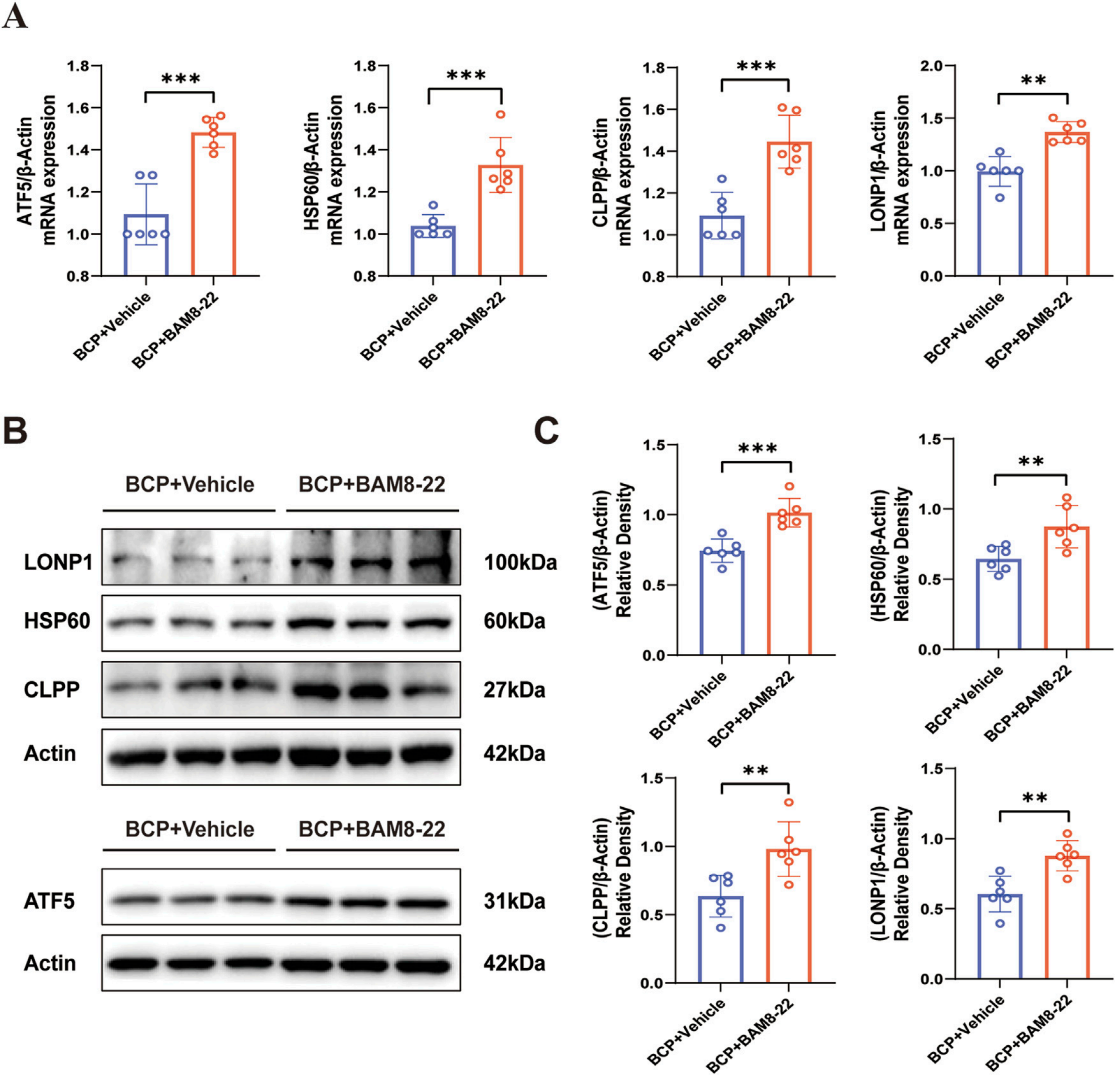
BAM8-22 mediates UPR^mt^ activity. **(A)** The mRNA expression profiles of UPR^mt^-associated markers (ATF5, HSP60, CLPP, and LONP1) in the ipsilateral spinal cord were quantitatively assessed using RT-PCR, comparing the treatment group with solvent controls. **(B)** Protein expression levels of ATF5, HSP60, CLPP, and LONP1 in the spinal cord were assessed by Western blotting in both experimental groups. **(C)** ATF5, HSP60, CLPP, and LONP1 quantitative analysis. (Independent t-test; *p < 0.05, **p < 0.01, ***p < 0.001 in comparison to the solvent group; n = 6 for each group). The mRNA expression profiles of UPR^mt^-associated markers (ATF5, HSP60, CLPP, and LONP1) in the ipsilateral spinal cord were quantitatively assessed using RT-PCR, comparing the treatment group with solvent controls.

UPR^mt^ serves as an essential cellular defense system that maintains mitochondrial protein homeostasis by upregulating molecular chaperones and proteases, which assist in either refolding or degrading misfolded proteins (Zhou et al., 2022). It has been demonstrated that GPCR and its downstream signaling pathways can activate UPR^mt^ contributing to mitochondrial homeostasis (Liu Y. et al., 2022). Therefore, when MrgC was activated, we looked into the impact of intrathecal BAM8-22 injection on spinal UPR^mt^. Six hours following the intrathecal injection of BAM8-22, the transcript levels of UPR^mt^-related genes (ATF5, HSP60, CLPP, and LONP1) were considerably higher than those of the solvent group (Figure 5A). Similarly, animals in the BAM8-22 group had significantly higher levels of UPR^mt^-related proteins (ATF5, HSP60, CLPP, and LONP1) in their spinal cord tissues (Figures 5B, C). These findings imply that intrathecal BAM8-22 injection improves mitochondrial activity in animals with bone cancer pain via raising UPR^mt^ expression levels in the spinal cord.

### Intervention of MrgC is involved in the expression changes of UPR^mt^-related proteins

**FIGURE 6:**
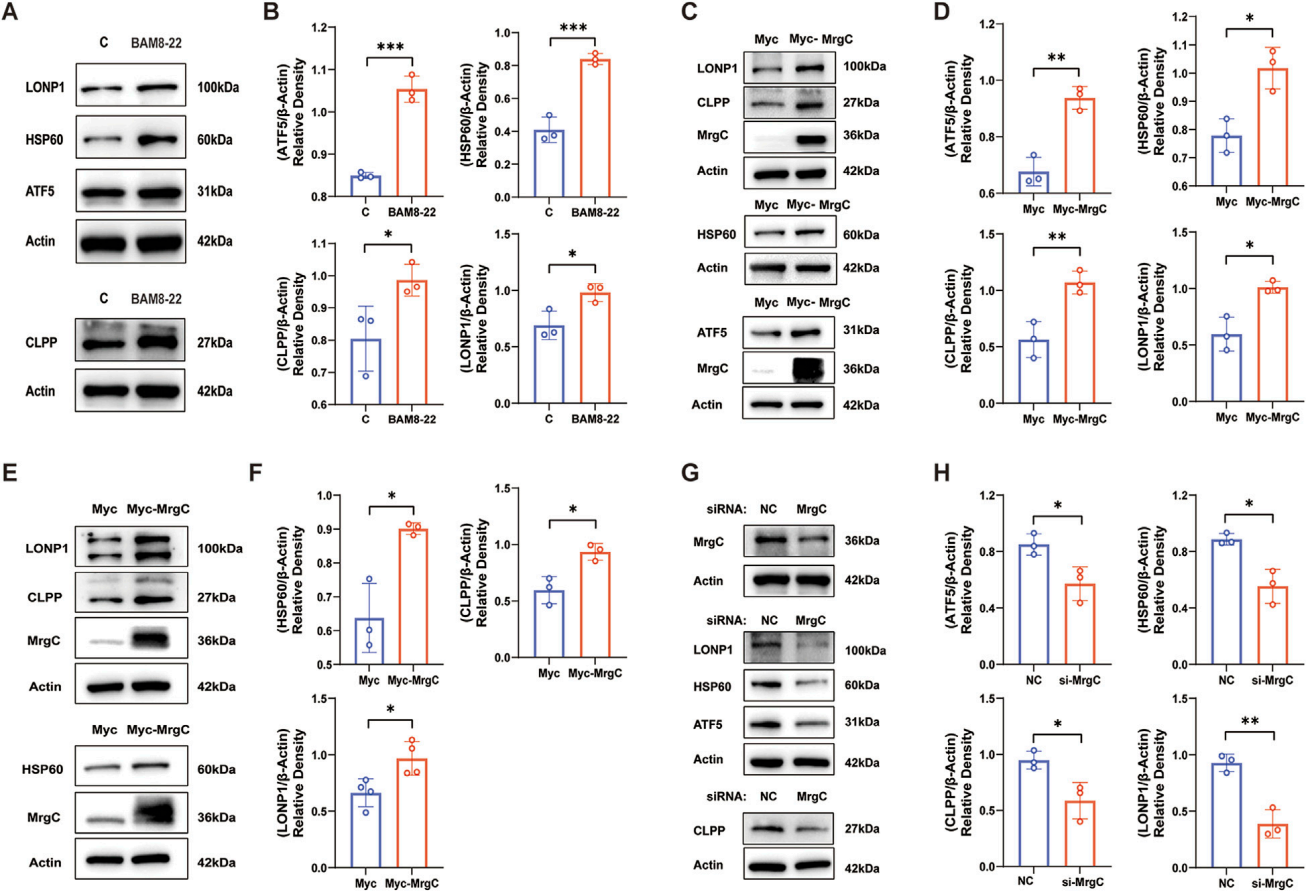
MrgC regulates cellular UPR^mt^ protein levels. **(A, B)** The expression levels of ATF5, HSP60, CLPP, and LONP1 as well as the relative expression histograms after BAM8-22 stimulation in N2a cells were evaluated by Western blotting analysis. **(C, D)** Western blotting was used to assess the expression levels and relative expression histograms of ATF5, HSP60, CLPP, LONP1, and MrgC after Myc-MrgC was overexpressed in N2a cells. **(E, F)** In 293T cells, Western blotting was similarly conducted after Myc-MrgC overexpression to analyze the expression levels and relative expression histograms of ATF5, HSP60, CLPP, LONP1, and MrgC. **(G, H)** Western blotting analysis was also performed to examine the protein changes of ATF5, HSP60, CLPP, LONP1, and MrgC, alongside relative expression histograms, following the silencing of MrgC in N2a cells. (Independent t-test, *p < 0.05, **p < 0.01, ***p < 0.001 compared to control; n = 3 per group).

To further validate the regulation of UPR^mt^-related proteins by MrgC, we examined the effects of both overexpression and knockdown of MrgC on UPR^mt^ signaling in Neuro-2a (N2a) cells. First, treatment of N2a cells with BAM8-22 resulted in upregulated protein expression of ATF5, HSP60, CLPP, and LONP1 (Figures 6A, B). In particular, we used a Myc-MrgC plasmid to transfect N2a cells. The Myc-MrgC group had considerably greater levels of expression of UPR^mt^-related proteins (ATF5, HSP60, CLPP, and LONP1) than the Myc-C group (Figures 6C, D). Similarly, transfection of the human Myc-MrgC plasmid into 293T cells resulted in a corresponding increase in UPR^mt^-related protein expression. (Figures 6E, F). Additionally, we knocked down MrgC expression using small interfering RNA (siRNA) targeting MrgC. The siMrgC group exhibited significantly reduced protein expression of ATF5, HSP60, CLPP, and LONP1 compared to the negative control (NC) group (Figures 6G, H). These findings imply that BAM8-22’s activation of MrgC influences the expression of proteins associated with UPR^mt^, contributing to the improvement of mitochondrial function.

## Discussion

Bone cancer pain (BCP), a debilitating neurological complication secondary to cancer-induced bone metastases and pathological bone remodeling, represents a critical clinical challenge in oncology palliative care. (Yang et al., 2023). Approximately 70% of cancer patients are reported to develop bone metastases, and BCP occurs in one-third of these patients (Chu et al., 2022). The majority of individuals suffering from BCP do not receive effective treatment, significantly diminishing their quality of life. Our findings revealed that BCP mice exhibited diminished mitochondrial membrane potential, increased ROS production, impaired ATP generation, and disrupted mitochondrial structure and functionality. Intrathecal administration of BAM8-22, an agonist of the MrgC receptor, was found to enhance mitochondrial function while alleviating pain behaviors in BCP mice. Activation of MrgC by BAM8-22 increases the expression of UPR^mt^-related proteins (ATF5, HSP60, CLPP and LONP1). Furthermore, the overexpression or knockdown of MrgC correspondingly influenced UPR^mt^ expression.

Mitochondria, known as the energy production centers of the cell, play a complex and critical role in pain regulation (Liu D. et al., 2022). An insufficient supply of energy to neurons can result from mitochondrial dysfunction, potentially altering the efficiency of pain signaling and resulting in enhanced or persistent pain signals (Luo et al., 2022; Willemen et al., 2023). Additionally, oxidative stress may exacerbate neuronal damage and further sensitize pain perception (Teixeira-Santos et al., 2020). In our study, we discovered that mice with bone cancer discomfort had enlarged mitochondria in their spinal cords, with the cristae structure either deformed or completely absent. These mice’s spinal cords showed increased ROS production, decreased mitochondrial membrane potential, and decreased ATP synthesis. In conclusion, mice with BCP showed negative effects on the mitochondria’s structure and function.

**FIGURE 7:**
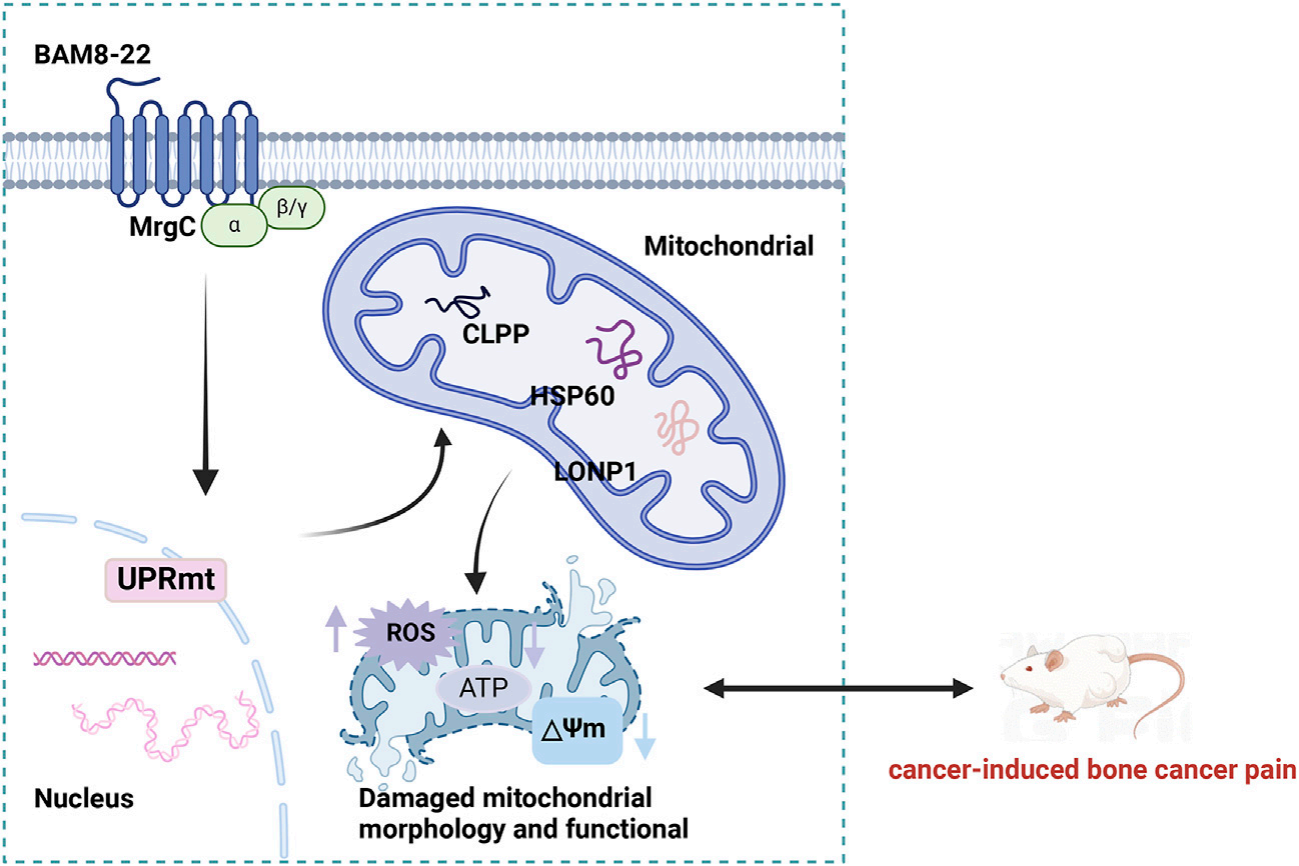
By modulating UPR^mt^ activity, BAM8-22 may reduce bone cancer discomfort in mice. ATF5, activating transcription factor 5; HSP60, heat shock protein 60; LONP1, Lon protease 1; CLPP, casein hydrolase; UPR^mt^, mitochondrial unfolded protein response. Figure Created with BioRender.com.

UPR^mt^ acts as an essential protective system for preserving mitochondrial function (Sutandy et al., 2023). When mitochondrial function is impaired, the inability to properly process unfolded proteins can result in mitochondrial dysfunction, negatively influencing the cell’s energy metabolism and antioxidant potential, and possibly initiating apoptosis (Lu et al., 2022). To avoid the accumulation of damaged proteins, enhance mitochondrial energy metabolism, mitigate the excess of ROS, and boost ATP production, UPR^mt^ regulates mitochondrial metabolic activities (Zhang B. et al., 2024). As a result, the proteins linked to UPR^mt^ are critical for sustaining mitochondrial protein homeostasis and ensuring the proper execution of mitochondrial functions (Torres et al., 2024). Recent studies indicate that SRZ-75, a G protein-coupled receptor, engages with Gαq in chemosensory ADL neurons. This engagement prompts the secretion of neuropeptides that activate the UPR^mt^ in peripheral tissues (Liu Y. et al., 2022). Another study demonstrated that FSHR-1, a G protein-coupled receptor, plays a crucial role in the autonomous induction of UPR^mt^ (Kim and Sieburth, 2020). Our research demonstrated that activating the MrgC receptor using BAM8-22 resulted in an increase in UPR^mt^-related protein levels, improved mitochondrial function, and decreased bone cancer pain in BCP mice (Figure 7). This study provides the first experimental evidence that the BAM8-22-MrgC signaling axis restores mitochondrial homeostasis through UPR^mt^ regulation, establishing a mechanistic foundation for developing UPR^mt^-targeted therapeutic strategies in bone cancer pain management.

Our study redefines the functional paradigm of MrgC in pain regulation, breaking through the traditional neuroinflammation-dominated framework of BCP mechanisms. Our study demonstrates that MrgC activation can regulate mitochondrial homeostasis in multiple ways, leading to mitochondrial structural and functional recovery. Importantly, we identified the direct transcriptional regulation of UPR^mt^ mediated by MrgC and rigorously validated it by siRNA knockdown and overexpression experiments, thus systematically elucidating the molecular dialogue between GPCR signaling and mitochondrial homeostasis. However, This study has several limitations. Intracellular signaling by G protein-coupled receptors involves a complex series of processes that trigger various cellular responses, primarily through the activation of three classes of G proteins (Gs, Gi, and Gq) and their downstream effectors (Wootten et al., 2018). Our investigation represents a preliminary exploration of the mechanisms by which mitochondrial function is enhanced following the activation of MrgC. The impact of MrgC activation on UPR^mt^-related proteins may encompass additional intracellular signaling processes. We plan to explore this aspect in future studies, with the goal of establishing a theoretical foundation for BCP management.

## Conclusion

BAM8-22 improves neuronal mitochondrial function by targeting spinal MrgC receptor to modulate UPR^mt^ activity in mice with bone cancer pain and participates in the development of bone cancer pain.

## Data Availability

The raw data supporting the conclusions of this article will be made available by the authors, without undue reservation.
