# Supplementary material for: BAM8-22 targets spinal MrgC receptors to modulate UPRmt activity in the mechanism of bone cancer pain
Source: Front Pharmacol. 2025 Mar 31;16:1575733. doi: 10.3389/fphar.2025.1575733 (PMC11994654; doi:10.3389/fphar.2025.1575733)
Supplement: Supplementary file 1 [file DataSheet1.DOCX]

Supplementary Material

**BAM8-22 improves mitochondrial function in mouse spinal cord and alleviates bone cancer pain in mice**

BAM8-22 is a specific agonist of the MrgC receptor. To investigate the effects of MrgC activation on mitochondrial function, we intrathecally injected BAM8-22 into mice on day 21 post-surgery and harvested spinal cord tissues 6 hours later to assess changes in mitochondrial function. Compared to the B+V group, the intrathecal injection of BAM8-22 resulted in a significant increase in spinal cord ATP levels (Figure S1A), a notable reduction in reactive oxygen species (ROS) levels (Figures S1B and C), and an enhancement in mitochondrial membrane potential (Figures S1D and E). Pain behavioral assessments indicated that, relative to pre-administration levels, 6 hours following BAM8-22 administration, there was a significant increase in the paw withdrawal mechanical threshold (PWMT) and a substantial decrease in the number of spontaneous flinches (NSF) (Figures S1F and G). In contrast, no significant changes in pain behavior were observed in the vehicle group (Figures S1F and G). In conclusion, BAM8-22 enhanced the structure and function of spinal cord mitochondria and alleviated bone cancer pain in mice under bone cancer pain (BCP) conditions.


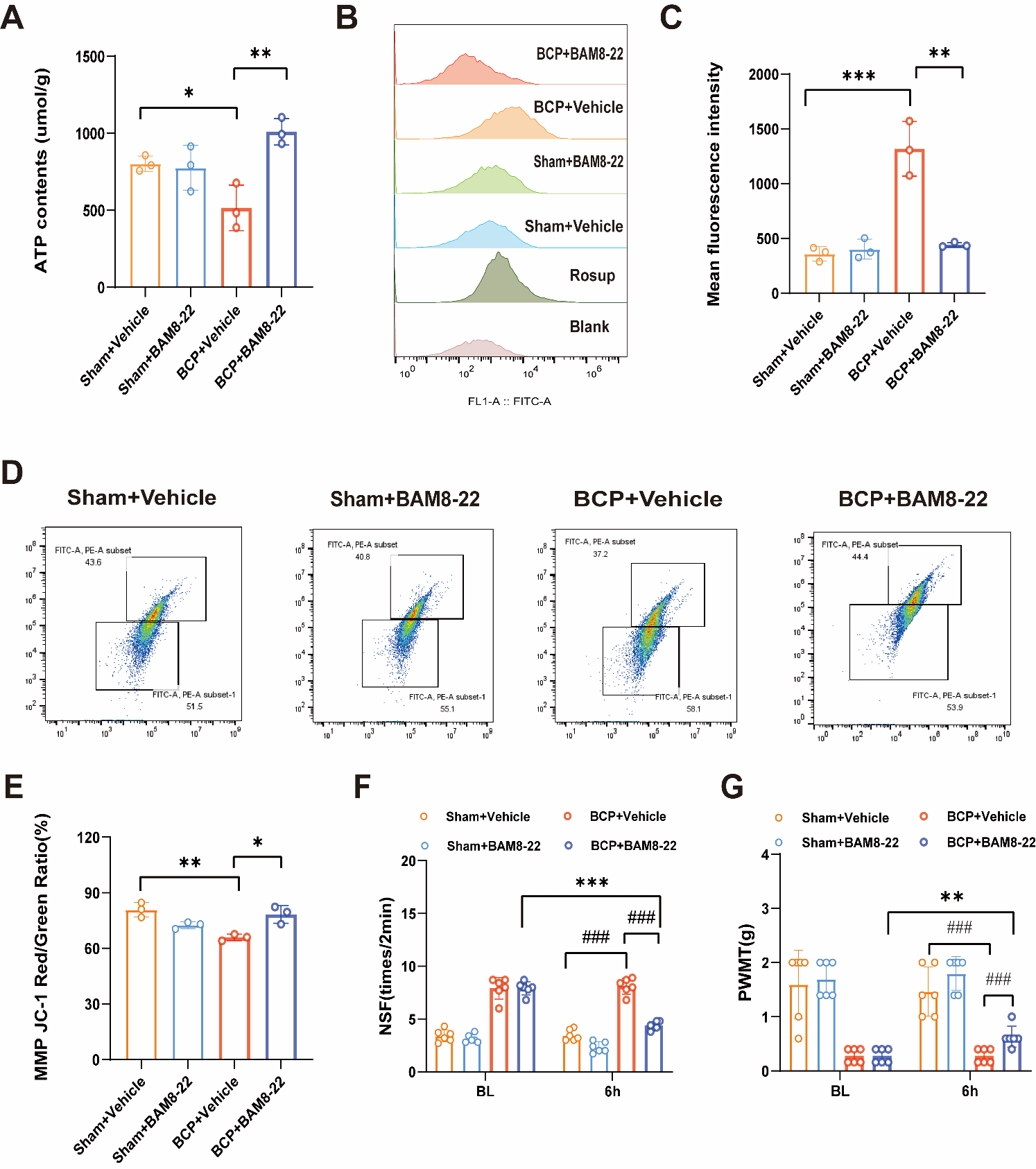


**Figure S1. Effect of BAM8-22 on mitochondrial function.**

**(A)** Mitochondrial ATP levels in the spinal cord are presented. **(B and C)** Flow histograms and quantitative plots illustrate the mean fluorescence intensity of mitochondrial reactive oxygen species (mtROS) in spinal cord tissue. **(D and E)** Flow scatter plots and quantitative maps depict mitochondrial membrane potential (MMP) in the spinal cord. **(F and G)** The paw withdrawal mechanical threshold (PWMT) and the number of spontaneous flinches (NSF) of mice were measured 21 days post-surgery and 6 hours following intrathecal injection of BAM8-22. Statistical analysis was conducted using an independent t-test, with significance levels indicated as * p < 0.05, ** p < 0.01, and *** p < 0.001, compared to the solvent group; n = 3 per group. Data are expressed as mean ± standard deviation.
